# Multifunctional *Bacillus* Strains for Integrated Fruit Rot Management and Improved Soil Phosphorus Availability in Longan Orchards

**DOI:** 10.3390/jof12080550

**Published:** 2026-07-23

**Authors:** Pisi Suksakol, Chananbhorn Thongrote, Tatiya Bansra, Kanlayawat Intha, Jirapinya Liamkraituan, Supuk Mahadtanapuk

**Affiliations:** 1 Program in Environmental Technology and Management, School of Energy and Environment, University of Phayao, 19 Moo 2 Maeka Subdistrict, Mueang District, Phayao 56000, Thailand; pisisuksakol@gmail.com; 2 Innovation and Technology Transfer Institute, University of Phayao, 19 Moo 2 Maeka Subdistrict, Mueang District, Phayao 56000, Thailand; chananbhorn.t@gmail.com; 3 Program in Biotechnology, School of Agriculture and Natural Resources, University of Phayao, 19 Moo Maeka Subdistrict, Mueang District, Phayao 56000, Thailand; ttiyabansra@gmail.com; 4 Department of Medical Sciences, Ministry of Public Health, 88/7 Tiwanon Road, Talat Khwan Subdistrict, Mueang Nonthaburi District, Nonthaburi 11000, Thailand; kanlayawat.i@dmsc.mail.go.th; 5 Sci Spec Co., Ltd., Head Office, 10 Kanchanapisek Road, Soi 0012 2nd Junction, Bangkae, Bangkok 10160, Thailand; jirapinyani@gmail.com

**Keywords:** *Bacillus* spp., *Lasiodiplodia pseudotheobromae*, phosphate solubilization, biological control, longan, plant-growth-promoting bacteria

## Abstract

Fruit rot caused by *Lasiodiplodia pseudotheobromae* poses a serious threat to longan (*Dimocarpus longan* Lour.) production. The present study evaluated antagonistic and phosphate-solubilizing bacteria for their potential use in environmentally responsible disease and soil management. Two complementary *Bacillus* isolates were selected. *Bacillus amyloliquefaciens* UPBA63 exhibited strong antagonistic activity against *L. pseudotheobromae*, whereas *Bacillus subtilis* SBP04 demonstrated superior phosphate-solubilizing and plant-growth-promoting traits. Their combined application provided complementary benefits for disease suppression and soil phosphorus availability. Biochemical assays and molecular identification using concatenated 16S rDNA and *gyrB* sequences assigned UPBA63 to *Bacillus amyloliquefaciens* and SBP04 to *B. subtilis*. Both isolates produced extracellular protease, cellulase, and amylase. UPBA63 significantly reduced fruit rot incidence in planta and during field trials, whereas SBP04 exhibited the greatest phosphate-solubilizing activity. Field application of UPBA63 and SBP04 combined with organic matter and a chemical fertilizer and fungicide at half the recommended doses reduced disease incidence to 15.40 ± 7.42% and improved yield relative to the untreated control. The integrated treatment combining *Bacillus* spp. with organic matter, half-rate chemical fertilizer, and half-rate fungicide resulted in a soil pH of 4.56, an organic matter content of 2.92%, and an available phosphorus of 149.25 mg kg^−1^. Multifunctional *Bacillus* strains demonstrated dual capacity for pathogen suppression and nutrient mobilization, supporting their potential application in sustainable disease control and soil fertility management in longan orchards.

## 1. Introduction

Longan (*Dimocarpus longan* Lour.) is an economically important fruit crop cultivated across tropical and subtropical regions. Its nutritional value and documented health-promoting properties contribute to strong consumer demand and commercial interest [1,2]. In Thailand, longan production constitutes a key component of the horticultural sector, supported by a substantial export market, particularly across Southeast Asia [3,4,5]. Nevertheless, fruit rot caused by *Lasiodiplodia pseudotheobromae* poses a serious threat to the sustainability and profitability of longan production. Infection by this fungal pathogen reduces fruit quality, postharvest longevity, and marketability, creating considerable economic risks for producers and exporters [6].

Longan orchards in northern Thailand, particularly in Chiang Mai, Lamphun, and Phayao Provinces, have experienced peel discoloration, fruit cracking, and fruit rot. Severe disease outbreaks have resulted in yield losses of up to 80% [7,8]. One notable outbreak occurred in 2002 in Chiang Kham District, Phayao Province, causing substantial economic damage. Later surveys conducted from 2017 to 2018 confirmed persistent disease incidence, especially in export-grade fruit, which must meet strict criteria for size and peel coloration [6]. Disease control depends mainly on chemical fungicides. Repeated and extensive fungicide use, however, has led to growing concern over chemical residue accumulation, resistant pathogen populations, and risks to environmental and human health. Given these constraints, sustainable and environmentally friendly disease control alternatives are needed, particularly biological control strategies [9].

In addition to disease pressure, declining soil fertility has emerged as a significant constraint in longan orchards in northern Thailand. The prolonged and excessive application of chemical fertilizers has contributed to soil degradation, thereby negatively affecting both fruit yield and quality. Phosphorus (P) is an essential macronutrient required for plant growth, energy transfer, and reproduction. However, a large proportion of phosphorus in soil exists in insoluble forms, particularly calcium-bound phosphates, which greatly limits its bioavailability to plants [5,7]. Thus, crops are often unable to efficiently utilize the phosphorus present in the soil. To overcome phosphorus deficiency, agricultural systems frequently rely on the intensive application of phosphate fertilizers. Although these practices are intended to enhance productivity, they often lead to nutrient imbalances and further deterioration of soil structure [8]. Longan production systems are especially vulnerable to these consequences due to their long-term reliance on synthetic fertilizers, which can reduce soil fertility and increase susceptibility to pests and diseases. The use of phosphate-solubilizing bacteria (PSB), hence, presents a promising strategy to enhance phosphorus availability, improve nutrient use efficiency, and reduce dependence on chemical inputs in sustainable horticultural systems.

Moreover, *Bacillus* species have drawn considerable research attention owing to their potential roles in biological disease control and plant growth promotion [10,11,12]. Certain *Bacillus* strains suppress plant pathogens through antagonistic activity, whereas others function as phosphate-solubilizing microorganisms capable of deriving phosphorus from insoluble phosphate compounds. Phosphate solubilization is driven mainly by organic acid production during microbial metabolism. Organic acid secretion lowers rhizosphere pH and dissolves mineral phosphates, releasing phosphorus in forms accessible to plants. Previous studies have linked the process principally to gluconic acid production and a substantial decline in rhizosphere pH [11].

Beneficial microorganisms have emerged as environmentally friendly alternatives to synthetic fungicides for plant disease management. In particular, *Bacillus* spp. can suppress phytopathogenic fungi through the production of antimicrobial metabolites and may also enhance plant defense responses. Biological control agents have shown promising potential for managing postharvest diseases, particularly when combined with complementary treatments to improve efficacy [13]. Recent attention has focused on microbial consortia and metabolite-based bioformulations, which may provide complementary or synergistic antifungal effects. The authors of [14], for example, demonstrated that microbial fermentates derived from *Lactiplantibacillus plantarum* and *Arthrospira platensis* inhibited several toxigenic fungi and reduced fungal infections in maize and lemon fruit. Integrated disease management strategies may further enhance disease suppression while reducing reliance on synthetic fungicides and the risk of fungicide resistance [9].

Among microbial biocontrol agents, *B. amyloliquefaciens* and *B. subtilis* are particularly promising due to their resilient endospores, which facilitate survival during storage and field application. Additionally, these species produce diverse antimicrobial metabolites and hydrolytic enzymes while simultaneously exhibiting plant-growth-promoting traits, including phosphate solubilization, phytohormone synthesis, and siderophore production. These multifunctional characteristics make them promising candidates for integrated disease management and sustainable crop production. In this context, the present study was conducted in collaboration with industrial and community stakeholders facing persistent disease challenges in longan orchards. Although *Bacillus* species, including *B. subtilis* and *B. amyloliquefaciens*, have demonstrated strong antagonistic activity against postharvest longan pathogens such as *L. pseudotheobromae* and *Pestalotiopsis* spp. [15], prior research has focused almost exclusively on postharvest disease suppression. To address this limitation, we evaluated two *Bacillus* isolates characterized by multifunctional, complementary biological functions: disease suppression and phosphate solubilization. Furthermore, we assessed their efficacy under field orchard conditions, establishing a framework for an integrated pre- and postharvest disease management strategy.

The objectives were (i) to isolate and identify bacterial strains exhibiting antagonistic activity against *L. pseudotheobromae*; (ii) to determine the effective concentration of selected antagonistic isolates for disease suppression; and (iii) to characterize phosphate-solubilizing isolates and evaluate their potential as microbial inoculants. Taxonomic identification was performed through morphological and molecular analyses, followed by an evaluation of mineral phosphate solubilization capacity. The antagonistic and nutrient-solubilizing characteristics of the chosen isolates were assessed to ascertain their potential as environmentally sound substitutes for traditional fungicides. This study is expected to contribute to the development of sustainable bio-based strategies for disease management and soil fertility improvement in commercial longan production.

## 2. Materials and Methods

### 2.1. Fungal Isolation and Scanning Electron Microscopy (SEM) Analysis

Longan fruits showing typical symptoms of fruit rot were collected from a commercial orchard in Phayao Province, Thailand. Infected tissues were excised from the lesion margins of the fruit peel and subjected to surface sterilization using 70% ethanol for 30 s, followed by rinsing in sterile distilled water and air-drying under aseptic conditions. The effectiveness of surface sterilization was verified by plating 100 µL of the final rinse water onto potato dextrose agar (PDA; Sigma-Aldrich, St. Louis, MO, USA). The absence of microbial growth after incubation confirmed successful surface sterilization before fungal isolation. Small tissue segments (approximately 3–5 mm^2^) were aseptically transferred onto potato dextrose agar (PDA) plates using the tissue transplantation technique described in [16]. The plates were incubated at 28 ± 2 °C for 7 days under ambient laboratory conditions. After the initial fungal colonies appeared, the fungi were subcultured, and pure cultures were obtained using the hyphal tip isolation method. Mycelium from each colony was aseptically transferred using a sterile needle onto fresh PDA medium to establish pure cultures. The purified isolates were maintained on PDA slants at 4 °C for subsequent morphological identification and pathogenicity tests. The isolated fungus was then evaluated for pathogenicity according to Koch’s postulates [17]. Each fungal isolate was inoculated onto ten healthy longan fruits, with three biological replicates. A mycelial plug (5 mm in diameter) taken from the actively growing margin of a 7-day-old culture was placed onto a wounded fruit surface. Sterile PDA plugs were used as the negative control. The inoculated fruits were incubated at 25 ± 2 °C under high humidity for 7 days. Disease severity was assessed by measuring the lesion diameter (mm) on each fruit using a digital caliper. Disease incidence (%) was calculated as the percentage of inoculated fruits showing typical symptoms. The isolate with the highest lesion diameter and disease incidence was selected for subsequent experiments. Then, morphological characterization and phylogenetic analyses based on the internal transcribed spacer (ITS), translation elongation factor 1-alpha (*TEF1-α*), and beta-tubulin (*tub2*) gene regions were performed to identify the potential causal agent of longan fruit rot disease [18]. Genomic DNA of representative isolates was extracted from 7-day-old aerial mycelium of pure cultures using the DNeasy Plant Mini Kit (Qiagen, Hilden, Germany). The three gene loci of the fungal isolates were amplified using the primer sets ITS4/ITS5, TEF1-688F/TEF1-1251R, and Bt2a/Bt2b, respectively [19,20,21]. The sequences obtained from the genes of the isolates were analyzed by comparing them against the NCBI GenBank database. The Molecular Evolutionary Genetic Analysis (MEGA12) software was used to construct the phylogenetic tree. A phylogenetic tree was constructed utilizing the maximum-likelihood method, and bootstrap testing was performed with 1000 replicates following the method described by Tamura et al. [22].

Microscopic analysis commenced with an examination of the infected regions using scanning electron microscopy (SEM; JEOL JSM-5910LV, Japan) to evaluate surface characteristics and detect fungal structures associated with disease development. Minute specimens of both the impacted and adjacent unblemished tissue were meticulously obtained. These specimens were then immediately fixed in a 2.5% (*v*/*v*) glutaraldehyde solution (Sigma-Aldrich, St. Louis, MO, USA), prepared in 0.1 M phosphate buffer (pH 7.2) (Sigma-Aldrich, St. Louis, MO, USA), and maintained at 4 °C for a duration of 12–24 h. After the initial fixation, the samples were rinsed three times in the same phosphate buffer (10 min per rinse) and post-fixed in 1% (*w*/*v*) osmium tetroxide solution (Sigma-Aldrich, St. Louis, MO, USA) for 1–2 h at room temperature. After that, the samples were rinsed again with phosphate buffer and then dehydrated using a series of ethanol solutions with increasing concentrations. Complete dehydration was achieved with absolute ethanol prior to critical point drying. The dried specimens were then attached to aluminum stubs using conductive carbon tape and sputter-coated with a thin layer of gold. Lastly, the prepared samples were examined and photographed using a scanning electron microscope operated at an accelerating voltage appropriate for biological specimens (e.g., 10–20 kV) according to standard procedures [23].

### 2.2. Isolation of Antagonistic Bacteria

Bacterial isolates were obtained from healthy longan fruits and leaves collected from orchards in Phayao Province, Thailand, and screened for antagonistic activity against the fungal pathogen using a dual-culture assay on potato dextrose agar (PDA), as described previously [24].

For the antagonism assay, a mycelial plug (5 mm diameter) taken from the margin of an actively growing fungal culture was placed at the center of a PDA plate. The bacterial isolate was streaked onto the same plate at approximately 2.0–2.5 cm from the fungal plug. Plates inoculated with the fungal plug alone served as the control treatment. Each treatment was performed in triplicate.

All plates were incubated at 28 ± 2 °C for 7 days. Fungal growth was measured by determining the radial extension (cm) of the colony toward and away from the bacterial streak. The degree of inhibition was calculated by comparing fungal radial growth in the presence of bacteria with that in the control plates. Variation among replicates was generally less than 2 mm.

The percentage inhibition of mycelial growth was calculated using the following equation:

(1) $$Inhibition\left(\%\right)=\;\frac{\left(R1-R2\right)}{R1}\;\times 100$$

where R1 and R2 represent the radial growth (cm) of the fungal colony in the control treatment and in the presence of the bacterial isolate, respectively. Isolates exhibiting significant inhibition were selected for further characterization. Percentage data were arcsine square-root-transformed before statistical analysis.

### 2.3. Isolation of Bacteria for Phosphate Solubilization

Rhizosphere soil samples were collected from longan orchards managed without chemical inputs. Samples were obtained from a depth of approximately 10–15 cm using a sterile soil auger, transferred into sterile polyethylene bags, and transported immediately to the laboratory for analysis [25].

To isolate the bacteria, 10 g of soil was suspended in 90 mL of sterile distilled water and homogenized by shaking at 150 rpm for 30 min. The suspension was serially diluted (10^−1^–10^−6^), and aliquots (100 µL) of appropriate dilutions were spread onto Pikovskaya’s agar (Himedia, Mumbai, India) containing insoluble tricalcium phosphate (Sigma-Aldrich, St. Louis, MO, USA) as the sole phosphorus source. Plates were incubated at 28 ± 2 °C for 3–5 days. Colonies that were surrounded by clear halo zones, which indicated phosphate solubilization, were selected and purified through repeated streaking on fresh plates to obtain pure cultures [26].

Purified isolates producing prominent halo zones were inoculated into nutrient broth (NB; Merck, Darmstadt, Germany) and incubated for 24 h on a temperature-controlled rotary shaker at 150 rpm and 28 ± 2 °C to generate starter cultures. A 1% (*v*/*v*) aliquot of each starter culture was then transferred to Pikovskaya’s broth and incubated at identical temperature and agitation settings for 72 h to assess phosphate-solubilizing activity. Uninoculated Pikovskaya’s broth constituted the control treatment. The procedure followed the original method reported in [27], later adopted extensively for screening phosphate-solubilizing microorganisms [28,29].

### 2.4. In Vitro Analysis of Plant-Growth-Promoting Traits and Extracellular Enzyme Production

Following incubation, selected bacteria were cultured and centrifuged at 5000 rpm for 20 min, and the supernatants were collected to determine soluble phosphorus concentration using the vanadomolybdenum yellow (phosphomolybdate) colorimetric method [30]. Absorbance was measured spectrophotometrically at the appropriate wavelength (e.g., 420 nm), and soluble phosphorus content was calculated using a standard calibration curve prepared with KH_2_PO_4_ (Merck, Darmstadt, Germany).

The bacterial isolates were tested for several plant-growth-promoting (PGP) traits, including indole-3-acetic acid (IAA) production, ammonia production, hydrogen cyanide (HCN) production, 1-aminocyclopropane-1-carboxylate (ACC) deaminase activity, and phosphate and zinc solubilization.

Indole-3-acetic acid (IAA) production was assessed in nutrient broth supplemented with 5 mM L-tryptophan (Sigma-Aldrich, St. Louis, MO, USA). Cultures were incubated for 48 h, followed by the addition of Salkowski reagent comprising 50 mL of 35% HClO_4_ (Merck, Darmstadt, Germany) and 1 mL of 0.5 M FeCl_3_ (Merck, Darmstadt, Germany). Absorbance was subsequently measured at 530 nm [31].

Zinc solubilization was evaluated on Bunt and Rovira medium containing 0.1% zinc oxide (Sigma-Aldrich, St. Louis, MO, USA) and zinc carbonate (Sigma-Aldrich, St. Louis, MO, USA) as an insoluble zinc source [32]. Phosphate solubilization was quantified using the ammonium phosphomolybdate method [33].

Bacterial cultures were assayed for ammonia production by first incubating them in a peptone water medium (Sigma-Aldrich, St. Louis, MO, USA) (peptone 1%, NaCl 0.5%) for 48 h at 37 °C in a shaker incubator and then adding 0.1 mL of Nessler’s reagent to detect ammonium accumulation, as indicated by the development of a yellow-brown color [31].

HCN production was analyzed by the picrate assay on nutrient agar plates amended with glycine (4.4 g/L) (Sigma-Aldrich, St. Louis, MO, USA) and filter paper impregnated with 0.5% picric acid solution (Sigma-Aldrich, St. Louis, MO, USA) prepared with 2% sodium carbonate (Merck, Darmstadt, Germany), followed by incubation at 37 °C for 24–48 h. A color change from orange-red to brown indicates HCN production [34].

ACC deaminase activity was analyzed by inoculating *Bacillus* spp. into nitrogen-free broth amended with 3 mM ACC (Sigma-Aldrich, St. Louis, MO, USA) as the sole source of nitrogen. Cultures were grown in tryptic soy broth (Sigma-Aldrich, St. Louis, MO, USA) for 24 h at 37 °C and centrifuged at 10,000 rpm for 10 min. Pellets were washed thrice with saline and re-suspended in 1 mL of saline. This suspension was spot-inoculated onto Burk’s agar medium supplemented with 3 mM ACC as the sole nitrogen source. Burk’s medium (Himedia, Mumbai, India) with 0.2% ammonium sulfate (Merck, Darmstadt, Germany) served as a positive control, and the same medium without ACC and ammonium sulfate was used as a negative control. Cultures were incubated for 7 days at 37 °C, and differences in growth patterns on ACC-amended plates compared to positive and negative controls were recorded [35].

Bacteria were evaluated for siderophore production using the chrome azurol S (CAS) shuttle assay, as described by Schwyn and Neilands [36]. All the bacterial cultures were grown in King’s B broth medium (Himedia, Mumbai, India)for 48 h at 37 °C on a rotary shaker (120 rpm) and then centrifuged at 8,000 rpm for 10 min. A total of 2 mL of CAS assay solution was added to 1 mL of the supernatant and incubated at room temperature in dark conditions. Simultaneously, a blank was prepared using King’s B broth medium and CAS assay solution.

Protease production was assessed by streaking bacteria on skim milk agar (Himedia, Mumbai, India) plates and incubating them at 37 °C for 24–48 h [37]. Proteolytic activity was indicated by the formation of clear halo zones surrounding the bacterial colonies [38].

Cellulase activity was evaluated by using carboxymethyl cellulose (CMC) agar (Himedia, Mumbai, India) plates. The bacterial strain was inoculated onto the plates and incubated at 37 °C for 48 h. The formation of clear halo zones around the colonies after staining with Congo red solution (Sigma-Aldrich, St. Louis, MO, USA) confirmed the presence of cellulase activity [39].

Amylase production was determined using a solid medium supplemented with soluble starch. A loopful of bacterial culture was inoculated onto the plates and incubated at 28 °C for 72 h. To detect starch hydrolysis, 3 mL of iodine solution was poured onto the surface of the agar plate. The appearance of a clear zone around the colony after 3 min indicated amylase activity [40].

### 2.5. Morphological, Biochemical, and Molecular Identification of the Selected Isolates

The bacterial isolates demonstrating the most significant antagonistic activity and phosphate-solubilizing capability underwent thorough morphological, biochemical, and molecular analyses. The selected bacterial isolates were identified based on morphological culture and biochemical characteristics as described in Bergey’s Manual of Determinative Bacteriology [41].

Genomic DNA was extracted from a fresh overnight culture using a commercial DNA extraction kit (Genomic DNA Mini Kit Blood/Cultured Cell) following the manufacturer’s instructions. The bacterial 16S rRNA gene was amplified using the universal primers 27F (5′-AGAGTTTGATCMTGGCTCAG-3′) and 1492R (5′-TACGGYTACCTTGTTACGACTT-3′) according to Lane (1991) [42]. The *gyrB* gene was amplified using the primers UP-1 (5′-GAAGTCATCATGACCGTGCTGCAYGCNGGNGGNAA-3′) and UP-2r (5′-AGCAGGGTACGGATGTGCGAGCCRTCNACRTCNGCRT-3′), as described by Yamamoto and Harayama (1995) [43]. PCR amplification was performed in a total reaction volume of 25 µL containing 12.5 µL of 2× PCR Master mix, 1 µL of each primer (10 µM), and 1 µL (20 ng) of genomic DNA template; sterile distilled water was added to the final volume. The PCR was conducted using a thermal cycle, adhering to these specific parameters: an initial denaturation phase at 95 °C for 5 min; 35 cycles of denaturation at 95 °C for 30 s, annealing at 55–58 °C for 30 s, and extension at 72 °C for 1 min; followed by a final extension at 72 °C for 10 min [43]. The amplified products were analyzed by electrophoresis on a 1% (*w*/*v*) agarose gel stained with a nucleic acid dye and visualized under UV illumination. The expected amplicon sizes were approximately 1500 bp for the 16S rRNA gene and 1200 bp for the *gyrB* gene [42,43].

PCR products were purified and subjected to Sanger sequencing by a commercial sequencing service. Molecular identification of bacterial isolates was performed based on the amplification and sequencing of two marker genes—the 16S rRNA and *gyrB* genes. The obtained nucleotide sequences were compared against sequences available in the NCBI database using the BLASTn (https://blast.ncbi.nlm.nih.gov/Blast.cgi; accessed on 21 May 2026) to determine their closest phylogenetic relatives and confirm the taxonomic identity of the isolates. Phylogenetic relationships were subsequently inferred using the maximum-likelihood method [44] based on the 16S rRNA and *gyrB* gene sequences with a bootstrap value of 5000 replications.

### 2.6. Antagonistic Activity Under in Planta Conditions

The efficacy of the antagonistic *Bacillus* strains against *L. pseudotheobromae* was evaluated on artificially inoculated fruit. For in planta assays, longan fruits were surface-disinfected; the fruits were washed and dried, and before inoculation, the surface of each longan fruit was wiped with 70% ethanol for 30 s and allowed to air-dry under aseptic conditions to reduce surface microbial contamination [15]. This procedure was intended to reduce epiphytic microorganisms while maintaining longan fruit viability for subsequent inoculation. To assess the curative potential of the selected *Bacillus* isolates, longan fruits were inoculated with *L. pseudotheobromae* at 1 × 10^6^ spores mL^−1^ by aseptic needle wounding to a depth of 2–3 mm. One hour later, bacterial suspensions were applied directly to the inoculation sites, and disease development was evaluated after incubation. Subsequently, the fruits were divided into treatment groups, and each group was sprayed with 20 mL of one of the selected bacterial concentrations (1 × 10^2^, 1 × 10^4^, 1 × 10^6^, 1 × 10^8^ CFU/mL). Treatments were arranged in a Completely Randomized Design (CRD), with ten replicates per treatment and ten fruits per replicate. Following application, fruits were incubated in a humidity-controlled chamber at 28 ± 2 °C for 7 days. Disease symptoms were evaluated daily, and the number of infected fruits was recorded at the end of the incubation period. Disease incidence was calculated as the percentage of infected fruits relative to the total number of fruits assessed. Disease incidence (%) was calculated as follows:

(2) $$Disease\;incidence\;(\%)=\;\frac{Number\;of\;infected\;fruits}{Total\;number\;of\;fruits\;assessed}\times 100$$

Disease symptoms were identified based on the characteristic symptoms of postharvest fruit rot caused by *L. pseudotheobromae* on longan fruit, as previously described in [6], and data were subjected to analysis of variance (ANOVA) using SPSS version 26. Percentage data were arcsine square-root-transformed prior to analysis. Means were compared using Tukey’s honestly significant difference (HSD) test at *p* < 0.05.

### 2.7. Evaluation of the Selected Phosphate-Solubilizing Bacterial Isolates Under Pot Conditions

Six-month-old grafted longan plants (*Dimocarpus longan* Lour. cv. E-Dor) were used in the pot experiment. Plants with uniform height and stem diameter were selected for the experiment. The grafted plants, approximately 60 cm in height, were transplanted into 10 L plastic pots containing a growing medium consisting of topsoil, cocopeat, and rice husk charcoal at a ratio of 2:4:3 (*v*/*v*/*v*) [45]. The growth medium was created by planting substrates in a greenhouse, and all plants received the same watering and care [28]. All plants were maintained under controlled greenhouse conditions with uniform temperature, relative humidity, and light throughout the experiment. Treatments were arranged in a Completely Randomized Design (CRD) with four replicates, and each replicate consisted of three longan plants.

Eight treatments were evaluated: Treatment 1 comprised an untreated control using distilled water; Treatment 2 consisted of organic matter alone; Treatments 3 and 4 consisted of *Bacillus* spp. strains UPBA63 and SBP04, respectively; Treatment 5 consisted of a mixture of strains UPBA63 and SBP04; Treatments 6 and 7 combined organic matter with strains UPBA63 and SBP04, respectively; and Treatment 8 combined organic matter with a mixture of strains UPBA63 and SBP04.

Well-decomposed organic matter derived from composted agricultural residues was used as the organic amendment in this study. The organic matter contained decomposed plant materials and organic nutrients suitable for improving soil fertility, water-holding capacity, and microbial activity in containerized plant production systems [46]. The organic amendment was incorporated into the potting medium at a rate of 500 g per pot prior to the first microbial application. The application rate was selected based on common organic amendment practices used in tropical fruit nursery systems and containerized plant production.

The selected phosphate-solubilizing bacterial isolates were cultured on nutrient agar (NA) at room temperature for 48 h to obtain pure colonies. A single colony was inoculated into nutrient broth (NB) and incubated to increase biomass. The bacterial suspension was adjusted spectrophotometrically to an optical density corresponding to 10^8^ CFU mL^−1^. Each bacterial suspension was applied near the root zone as a soil drench at a volume of 100 mL per pot, together with the organic matter amendment. Treatments were applied every two weeks for a total of 12 applications throughout the experimental period. At the end of the experiment, five soil subsamples were collected from each pot and combined into one composite sample prior to chemical analysis. Soil pH, organic matter content, and available phosphorus were determined using standard soil analysis procedures reported in [47]. Soil organic matter content was quantified using the Walkley–Black dichromate oxidation method [48].

Data were subjected to analysis of variance (ANOVA) using SPSS version 26. Means were compared using Tukey’s honestly significant difference (HSD) test at *p* < 0.05.

### 2.8. Evaluation of a Multi-Strain Bacillus Formulation Under Field Conditions

A longan orchard with a documented history of *L. pseudotheobromae* infection was used to evaluate the formulation under field conditions [24].

**Figure 1:**
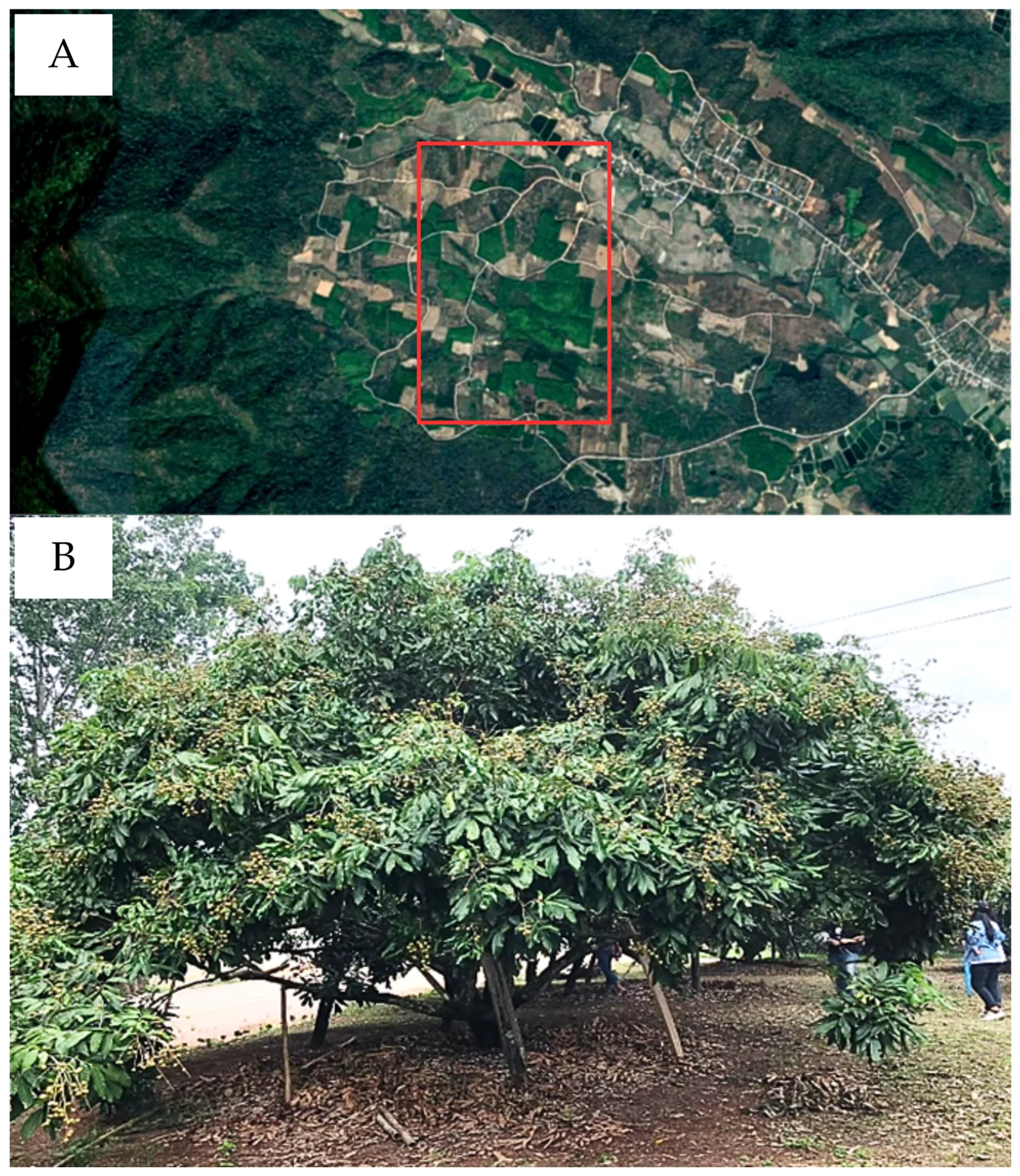
Location of the study area in Ban Tam Subdistrict, Phayao Province (red box). (**A**) Geographic coordinates (19°14′25.0” N 99°44′45.5” E); (**B**) the phenotypic characteristics of the longan tree used in the experiment.

The field trials were conducted in a commercial orchard using healthy seven-year-old longan plants (*Dimocarpus longan* Lour. cv. E-Dor), planted at a spacing of 6 × 6 m, with an average canopy radius of approximately 2.5–3 m (Figure 1B). One untreated buffer tree was maintained between adjacent experimental units to minimize microbial drift and spray interference. The longan leaves and soil samples were plated on a medium to confirm inoculum presence before trial initiation. Standard horticultural practices were followed, including pre-emergent herbicide application prior to planting, mechanical and manual weed control, and overhead irrigation (two to three times per week). A single field experiment was conducted using a randomized complete block design (RCBD) comprising seven treatments and four replications, with five longan trees assigned to each replication. The experiment was initiated in September 2023 with orchard preparation, including fertilizer application and tree management, followed by treatment application and disease evaluation in August 2024. Although the study spanned two calendar years because of the longan production cycle, all data were obtained from a single experimental trial. Experimental blocks were established based on similarities in tree vigor, canopy size, and orchard topography to reduce environmental variation across the experimental area. Each experimental unit comprised adjacent longan trees of uniform age and comparable growth characteristics. The seven treatments consisted of (1) an untreated control receiving water only, (2) organic matter alone, (3) a combined application of *Bacillus* spp. strains UPBA63 and SBP04, (4) strains UPBA63 and SBP04 combined with organic matter, (5) strains UPBA63 and SBP04 combined with organic matter and a chemical fertilizer and fungicide at half the recommended doses, (6) organic matter combined with a chemical fertilizer and fungicide at half the recommended doses, and (7) organic matter combined with a chemical fertilizer and fungicide at the full recommended doses.

The bacterial suspension containing strains UPBA63 and SBP04 was prepared at a final concentration of 10^8^ CFU mL^−1^ (cell density was adjusted spectrophotometrically and confirmed by serial dilution plating) and applied around the root zone of each longan tree at a rate of 2 L per tree. Foliar application was also performed by spraying approximately 1 L of the microbial suspension onto the canopy until runoff. The suspension was applied at two-week intervals throughout the experimental period. Organic matter amendment was applied once at a rate of 5 kg tree^−1^ around the soil surface beneath the canopy drip line to the first microbial application. A chemical fertilizer consisting of N, P, and K, together with supplementary nutrients, was applied following the conventional fertilization program commonly adopted by commercial longan growers in northern Thailand [49].

Difenoconazole was used as the chemical control treatment because it is commonly applied by longan growers for disease management. The fungicide was applied at the manufacturer’s recommended label rate throughout the experiment for the full-dose treatment, while the half-dose treatment received 50% of the recommended rate. All treatments were applied twice monthly for six consecutive months prior to harvest; they were applied under similar environmental conditions and at approximately the same time of day for each application event. Fruit yield was recorded at harvest and expressed as kg tree^−1^ by randomly collecting longan fruits from each treatment, and three randomly selected trees from each replication were used for analysis.

Disease incidence (%) was evaluated by randomly collecting longan fruits from each treatment at harvest. Fruits from three randomly selected trees from each replication (20 fruits per replicate) were cleaned with sterile distilled water, air-dried at room temperature, and placed in plastic boxes lined with moist sterile tissue paper to maintain high relative humidity. The boxes were sealed and incubated at 25–28 °C for 7 days to promote the development of symptoms caused by *L. pseudotheobromae*, following the method described in [6]. Disease incidence was calculated as the percentage of infected fruits showing visible disease symptoms (the calculation was performed according to the method described in Section 2.6). The pathogen was re-isolated from symptomatic tissues and morphologically identified to confirm disease etiology. Soil samples were collected from the upper 0–15 cm soil layer beneath the canopy drip line at the end of the experiment. Composite soil samples were obtained from four directions around each experimental unit and homogenized prior to analysis. Soil pH, organic matter content, and available phosphorus were determined according to standard soil analysis procedures [47]. Organic matter content was analyzed using the Walkley–Black dichromate oxidation method [48].

Data were subjected to analysis of variance (ANOVA) using SPSS version 26. Percentage data were arcsine square-root-transformed prior to analysis. Means were compared using Tukey’s honestly significant difference (HSD) test at *p* < 0.05.

## 3. Results

### 3.1. Fungal Isolation and Scanning Electron Microscopy (SEM) Analysis

A field experiment was conducted from September 2023 to August 2024 at 19°14′25.0” N 99°44′45.5” E (Figure 1). Monthly rainfall and temperature data in the orchard were obtained from the Thai Meteorological Department (TMD) weather summary reports for the Phayao meteorological station. During the study period, the average monthly rainfall was 126.92 mm, while temperatures ranged from 21.9 to 33.6 °C [50].

A total of 50 fungal isolates were obtained from symptomatic longan fruits and evaluated for pathogenicity under both controlled moisture chamber conditions (relative humidity: 90–95%; temperature: 23–33 °C) and orchard conditions. Among the seven fungal isolates tested, isolate UPL1 produced the largest mean lesion diameter, causing significant fruit rot compared with the sterile distilled water control (*p* < 0.05), and was therefore selected as the most virulent isolate for subsequent antagonism and biocontrol experiments. The disease incidence induced by isolate UPL1 was 90.00 ± 7.07, compared with 00.00 ± 0.00 in the control treatment.

The fungal isolate UPL1 was observed on PDA at 28 ± 2 °C for 7 days. Colonies were initially white with abundant fluffy aerial mycelia and became gray with age; they rapidly covered the entire Petri plate within 3–5 days. As the culture aged, the colony color gradually changed from grayish white to dark gray and eventually black on the reverse side of the plate. Microscopic observation revealed that the hyphae were septate, branched, and hyaline during the early growth stage. Pycnidia developed on PDA after incubation and appeared dark brown to black and solitary. Conidiogenous cells were hyaline, smooth-walled, and cylindrical. Immature conidia were hyaline, aseptate, thick-walled, and ellipsoidal to ovoid in shape. Mature conidia became dark brown and formed characteristic longitudinal striations. The conidia measured 30.0–32.0 × 18.0–20.0 µm. The morphological characteristics of the isolate were consistent with previously reported descriptions of *L. pseudotheobromae*, supporting its identification as the causal agent of longan fruit rot disease [6,21].

**Figure 2:**
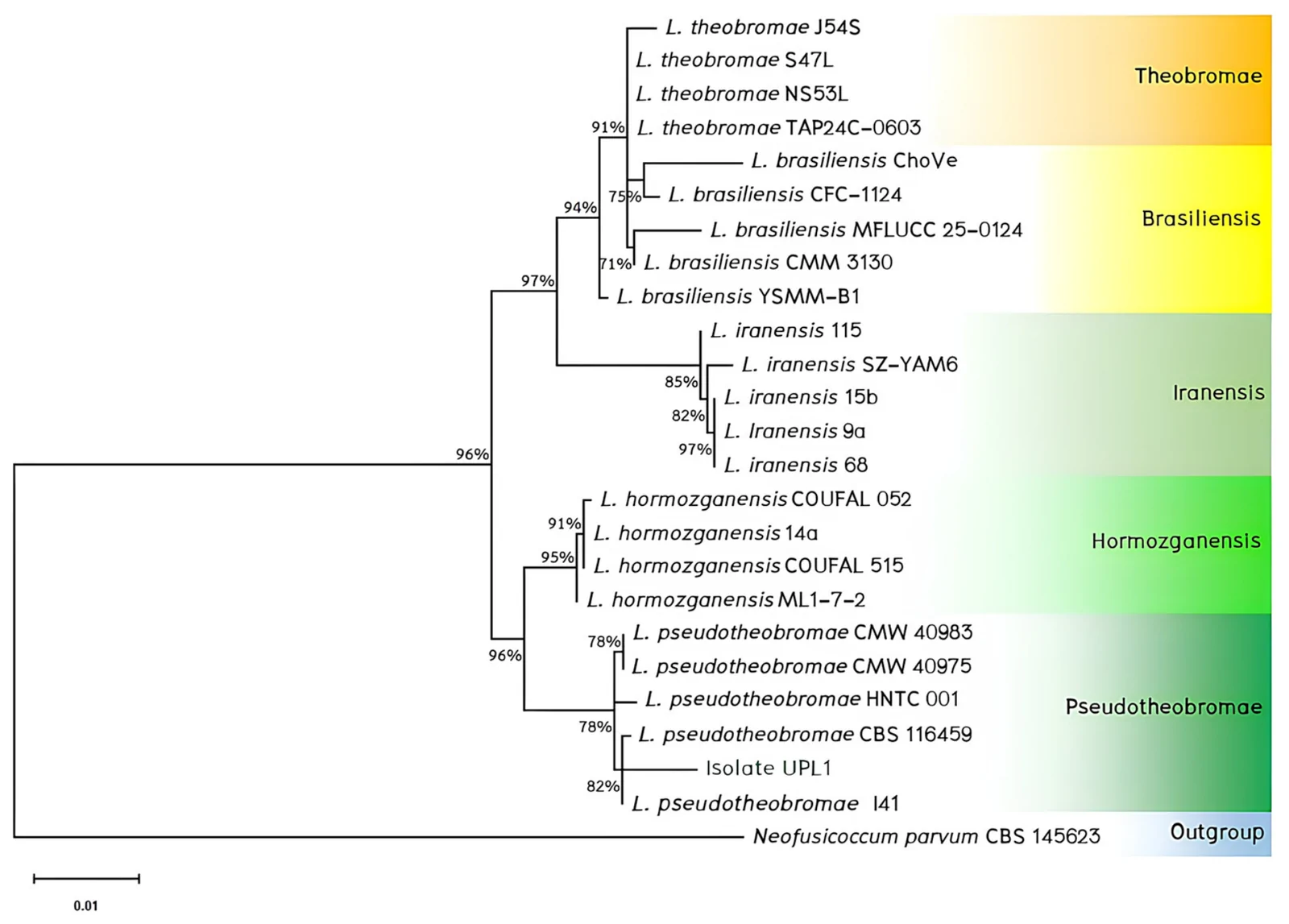
Phylogenetic tree derived from maximum-likelihood analysis based on combined sequence data of three loci (ITS, *TEF1-α*, and β-tubulin) of *Lasiodiplodia* species. *Neofusicoccum parvum* CBS 145623 was used as the outgroup. Numbers above branches represent bootstrap support values (%) based on 5000 replicates, and only values ≥ 70% are shown. Scale bar = 0.01 substitutions per site. Isolate UPL1 obtained in this study is highlighted in green.

Pathogenicity was further confirmed by re-inoculation of the isolate onto healthy longan fruits. Typical fruit rot symptoms developed within 3–5 days after inoculation, whereas control fruits remained symptomless. In line with Koch’s postulates, the fungus was re-isolated from symptomatic tissues of the inoculated fruits and identified based on its colony morphology and microscopic characteristics, which were consistent with those of the original isolate. No fungal pathogen was recovered from the control fruits. Subsequently, molecular identification was performed to confirm the species of the fungus. The internal transcribed spacer (ITS), translation elongation factor 1-alpha (*TEF1-α*), and beta-tubulin (*tub2*) gene regions were amplified and sequenced using an automated DNA sequencer (First BASE Laboratories Sdn. Bhd., Selangor, Malaysia). The resulting consensus sequences were obtained from three loci: ITS (544 bp; ITS1/ITS4), β-tubulin (459 bp; Bt2a/Bt2b), and *TEF1-α* (463 bp; TEF1-688F/TEF1-1251R). The sequences were subsequently analyzed using the Basic Local Alignment Search Tool (BLAST), and phylogenetic relationships were inferred based on the sequence alignment with reference sequences retrieved from the NCBI database. BLASTn analysis of the NCBI GenBank database revealed that isolate UPL1 showed high sequence similarity to *L*. *pseudotheobromae* reference isolates. Phylogenetic analysis clustered isolate UPL1 within the *L. pseudotheobromae* clade together with reference isolates CMW 40983 (ITS; Accession no. KP872340.1, β-tubulin; Accession no. KP872399.1, *TEF1-α*; Accession no. KP872369.1), CMW 40975 (ITS; Accession no. KP872332.1, β-tubulin; Accession no. KP872391.1, TEF1-α; Accession no. KP872361.1), HNTC 001 (ITS; Accession no. MK358285.1, β-tubulin; Accession no. MK419022.1, *TEF1-α*; Accession no. MK398313.1), CBS 116459 [21] (ITS; Accession no. EF622077.1, β-tubulin; Accession no. EU673111.1, *TEF1-α*; Accession no. EF622057.1), and I41 [51] (ITS; Accession no. MK693210.1, β-tubulin; Accession no. MK693701.1, *TEF1-α*; Accession no. MK693706.1), with bootstrap support values ranging from 78% to 82%. These results indicate that isolate UPL1 belongs to the genus *Lasiodiplodia* and is closely related to *L. pseudotheobromae* (Figure 2).

**Figure 3:**
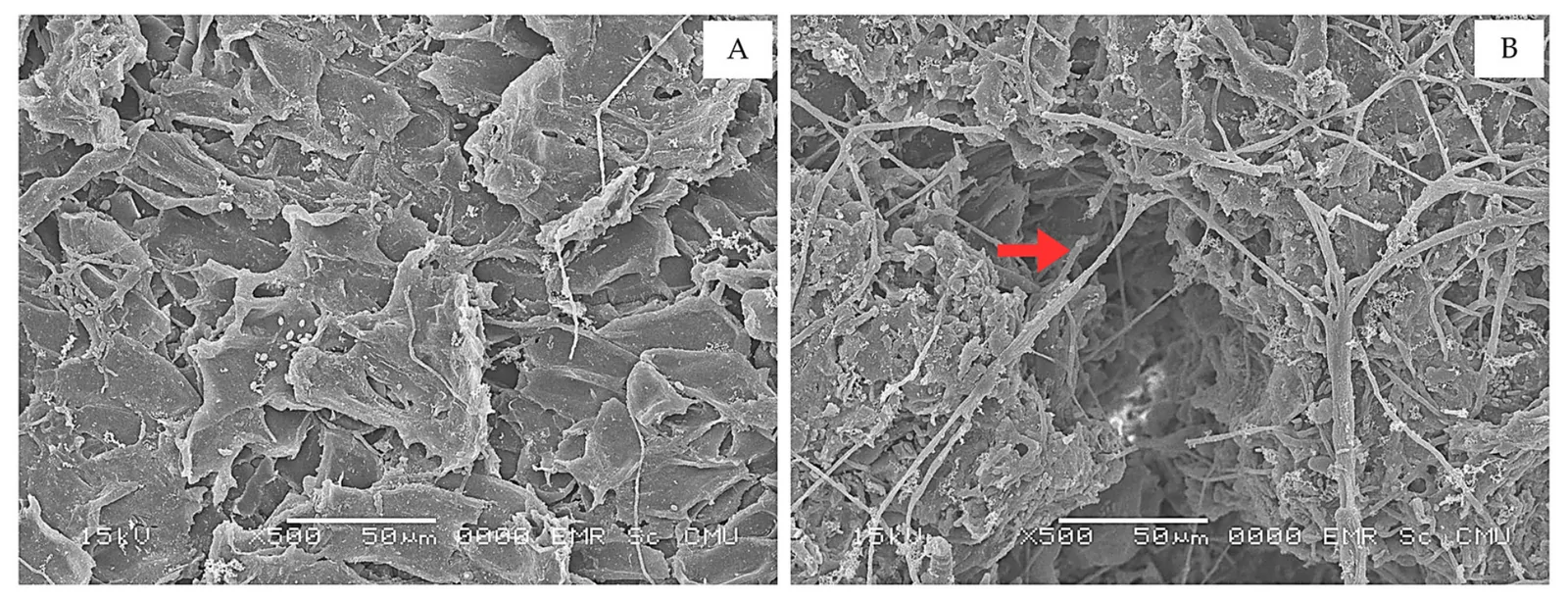
Scanning electron microscopy (SEM) observations of healthy and diseased longan fruit peels. (**A**) SEM micrograph of healthy peel tissue (scale bar = 50 µm), exhibiting an intact and smooth epidermal surface; (**B**) SEM micrograph of symptomatic peel tissue (scale bar = 50 µm), showing fungal mycelial colonization (red arrows).

In contrast, the affected tissues showed significant structural damage, including cracks in the outer layer and widespread fungal growth on the fruit surface (Figure 3). The presence of hyphae, which were seen growing through the cracked outer layer, indicated that the infection was active and that the tissue was being invaded.

Scanning electron microscopy (SEM) analysis revealed clear differences in the microscopic structures of healthy and infected peel tissues. The surface of healthy longan peel showed an intact and smooth epidermal layer without visible cracking (Figure 3A). In contrast, affected tissues showed significant structural damage, including epidermal fissures, extensive fungal mycelial colonization on the fruit surface, and hyphal penetration through the cracked epidermal layer, indicating active infection and tissue invasion (Figure 3B).

### 3.2. Isolation of Antagonistic Bacteria

With the aim of reducing reliance on chemical fungicides, more than 300 bacterial isolates obtained from longan fruits and soil were screened for antagonistic activity against *L. pseudotheobromae* using the dual-culture assay described previously.

Among the tested isolates, strain UPBA63 exhibited the strongest antifungal activity, significantly suppressing fungal radial growth by more than 70% compared with the control (*p* < 0.05). After 7 days of incubation, inhibition of mycelial growth was quantified based on the reduction in colony diameter, and results were expressed as percentage inhibition.

**Figure 4:**
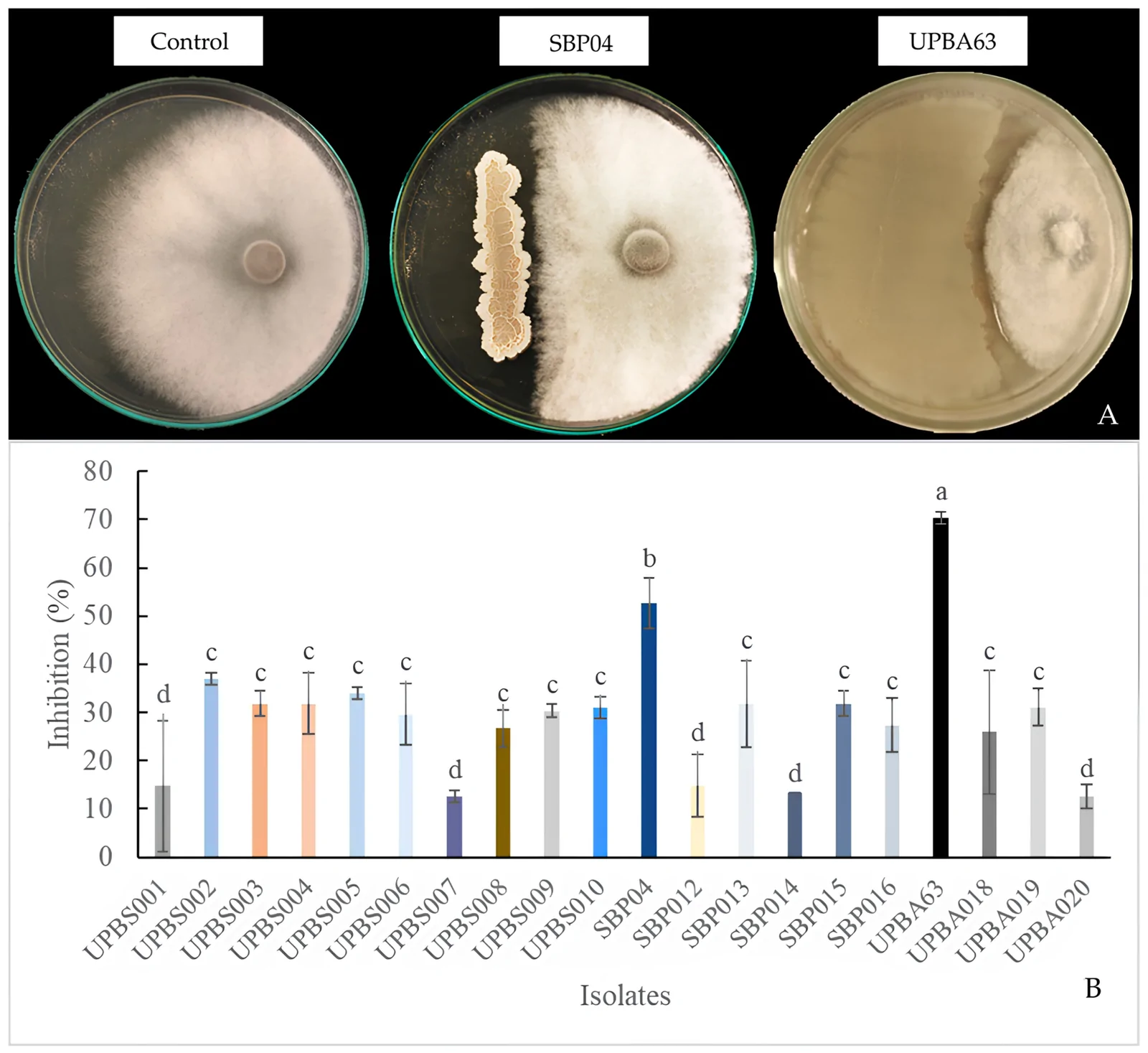
Results of a dual-culture assay for antagonistic activity of selected bacterial isolates against *L. pseudotheobromae* on PDA. (**A**) Representative dual-culture plates showing *L. pseudotheobromae* co-cultured with the indicated bacterial isolates (SBP04, UPBA63), together with the pathogen-only control, after 7 days of incubation. (**B**) Percentage inhibition of mycelial growth of *L. pseudotheobromae* by the selected bacterial isolates. Data were analyzed using a one-way analysis of variance (ANOVA). Different lowercase letters indicate significant differences among isolates according to Tukey’s honestly significant difference (HSD) test at *p* < 0.05. Percentage data were arcsine square-root-transformed before analysis. Original (untransformed) means are presented.

Isolate UPBA63 showed the highest inhibition rate (71.37 ± 1.28%), followed by isolate SBP04 (52.60 ± 5.23%). All selected isolates demonstrated statistically significant suppression of fungal growth compared with the untreated control (Figure 4).

### 3.3. Isolation of Bacteria for Phosphate Solubilization

Phosphate-solubilizing bacteria (PSB) were isolated from soil samples collected in a longan orchard. Soil suspensions were initially plated on the medium using the spread plate technique to obtain culturable bacterial populations. A total of 158 bacterial isolates were recovered.

**Figure 5:**
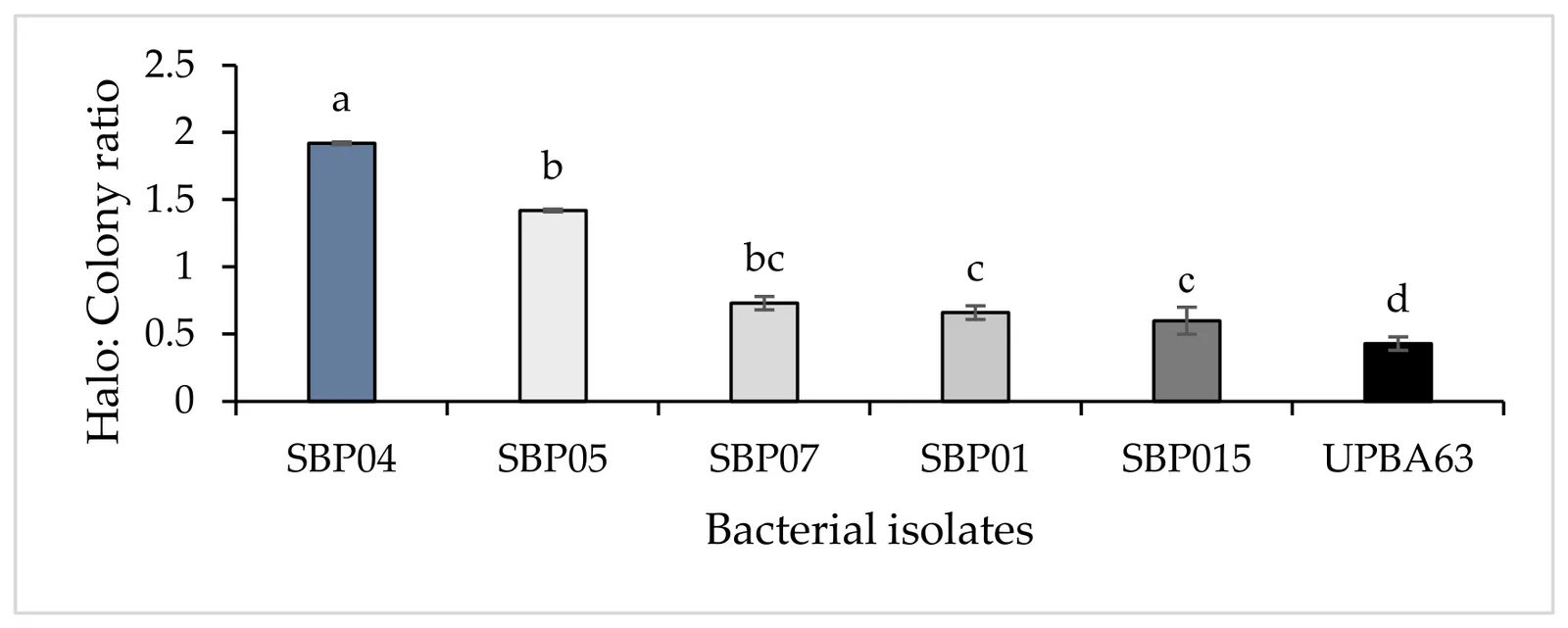
Phosphate-solubilizing ability of bacterial isolates determined by the halo-to-colony ratio on Pikovskaya’s agar, evaluating soluble phosphorus. Data were analyzed using a one-way analysis of variance (ANOVA). Different lowercase letters indicate significant differences among isolates according to Tukey’s honestly significant difference (HSD) test at *p* < 0.05.

Preliminary screening on Pikovskaya’s agar identified six isolates that produced clear halo zones surrounding their colonies, indicating phosphate solubilization activity (Figure 5). These isolates were designated SBP04, SBP05, SBP07, SBP01, SBP015, and UPBA63.

Phosphate-solubilizing efficiency was evaluated by calculating the halo-to-colony diameter ratio. Significant differences were observed among the isolates (*p* < 0.05). The ratios were 1.92 ± 0.01 (SBP04), 1.42 ± 0.01 (SBP05), 0.73 ± 0.05 (SBP07), 0.66 ± 0.05 (SBP01), 0.60 ± 0.10 (SBP015), and 0.43 ± 0.05 (UPBA63) (Figure 5).

### 3.4. In Vitro Analysis of Plant-Growth-Promoting Traits and Extracellular Enzyme Production

The plant-growth-promoting traits of the selected bacterial isolates are summarized in Table 1. Both isolates exhibited multiple beneficial characteristics associated with plant growth promotion. Isolate SBP04 showed higher phosphate-solubilizing activity (90.12 mg L^−1^) compared with UPBA63 (77.52 mg L^−1^) and produced a greater amount of indole-3-acetic acid (69 µg mL^−1^). Both isolates could produce ammonia and siderophores; however, SBP04 demonstrated stronger activity (+++) than UPBA63 (++). Zinc solubilization was not observed in either isolate. Overall, these results indicate that both bacterial isolates possess several plant-growth-promoting traits, with SBP04 exhibiting stronger biochemical activities (Table 1). In addition, the production of extracellular enzymes by the tested *Bacillus* isolates is reported in Table 2. The highest protease, amylase, and cellulase enzyme production was observed in UPBA63, followed by SBP04.

### 3.5. Morphological, Biochemical, and Molecular Identification of the Selected Isolate

In the dual-culture assay used to evaluate antagonistic activity against *L. pseudotheobromae*, isolate UPBA63 exhibited the highest percentage of mycelial growth inhibition and was therefore selected for further characterization. Among phosphate-solubilizing isolates, SBP04 demonstrated the highest halo-to-colony ratio on Pikovskaya’s agar. Statistical analysis confirmed that both isolates differed significantly from the other tested isolates at a 95% confidence level (*p* < 0.05). The two selected isolates were identified based on morphological characteristics, conventional biochemical assays, and molecular techniques. The antagonistic isolate UPBA63 was identified as *B. amyloliquefaciens*, whereas SBP04 was identified as *B. subtilis* (Table 3).

**Figure 6:**
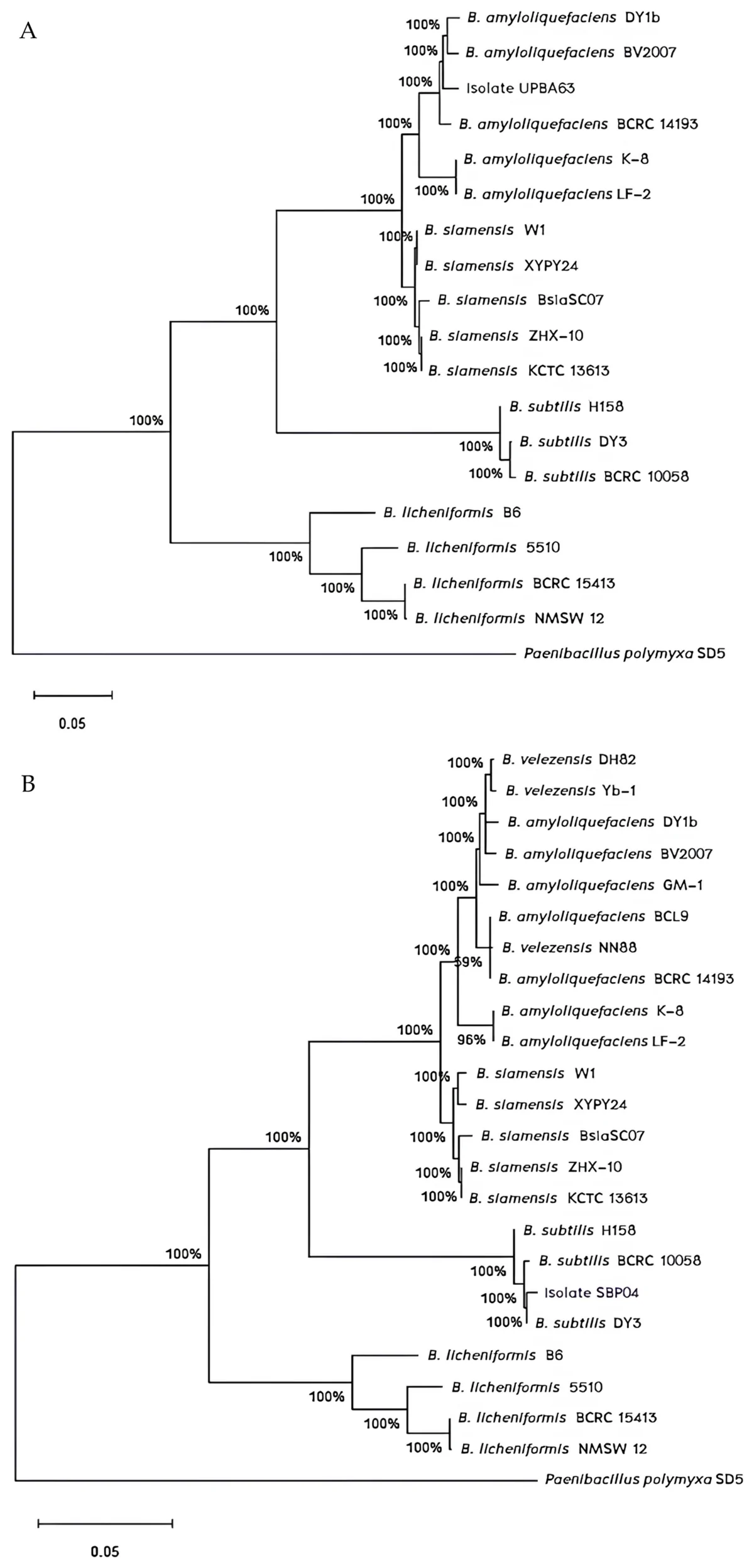
Phylogenetic trees derived from maximum-likelihood analysis based on combined sequence data of 16S rRNA and *gyrB* gene regions of *Bacillus* species. (**A**) Phylogenetic placement of isolate UPBA63 within the *B. amyloliquefaciens* clade. (**B**) Phylogenetic placement of isolate SBP04 within the *B. subtilis* clade. *Paenibacillus polymyxa* SD5 was used as the outgroup in both analyses. Numbers above branches represent bootstrap support values (%) based on 5000 replicates, and only values ≥ 70% are shown. Scale bar = 0.05 substitutions per site. Isolates obtained in this study are indicated as Isolate UPBA63 and Isolate SBP04.

The PCR products were purified and sequenced using an automated DNA sequencer (First BASE Laboratories Sdn. Bhd., Selangor, Malaysia). The obtained sequences were analyzed using the Basic Local Alignment Search Tool (BLAST), and phylogenetic relationships were inferred from 16S rRNA and *gyrB* gene sequences using the maximum-likelihood method with a bootstrap value of 5000 replications. Phylogenetic analyses based on both gene regions placed the two isolates within the genus *Bacillus* and clustered them with their respective reference strains. Isolate UPBA63 showed 100% sequence similarity to *Bacillus amyloliquefaciens* DY1b [52](16S rRNA; Accession no. KY290588.1, *gyrB*; Accession no. KY315726.1), BV 2007 (16S rRNA; Accession no. MT613661.1, *gyrB*; Accession no. MT670045.1), BCRC 14193 [53] (16S rRNA; Accession no. EF433408.1, *gyrB*; Accession no. DQ309309.1), K−8 (16S rRNA; Accession no. MT296780.1, *gyrB*; Accession no. MT296781.1), and LF−2 (16S rRNA; Accession no. MW418527.1, *gyrB*; Accession no. MW418529.1), whereas isolate SBP04 exhibited 100% sequence identity to *Bacillus subtilis* DY3 (16S rRNA; Accession no. KY290590.1, *gyrB*; Accession no. KY315728.1), H158 [54] (16S rRNA; Accession no. MT348555.1, *gyrB*; Accession no. MT359893.1), and BCRC 10058 [55] (16S rRNA; Accession no. DQ993674.1, *gyrB*; Accession no. DQ309306.1). The phylogenetic trees further confirmed the taxonomic placement of UPBA63 within the *B. amyloliquefaciens* clade and SBP04 within the *B. subtilis* clade, both supported by strong bootstrap values (Figure 6).

### 3.6. Antagonistic Activity Under in Planta Conditions

**Figure 7:**
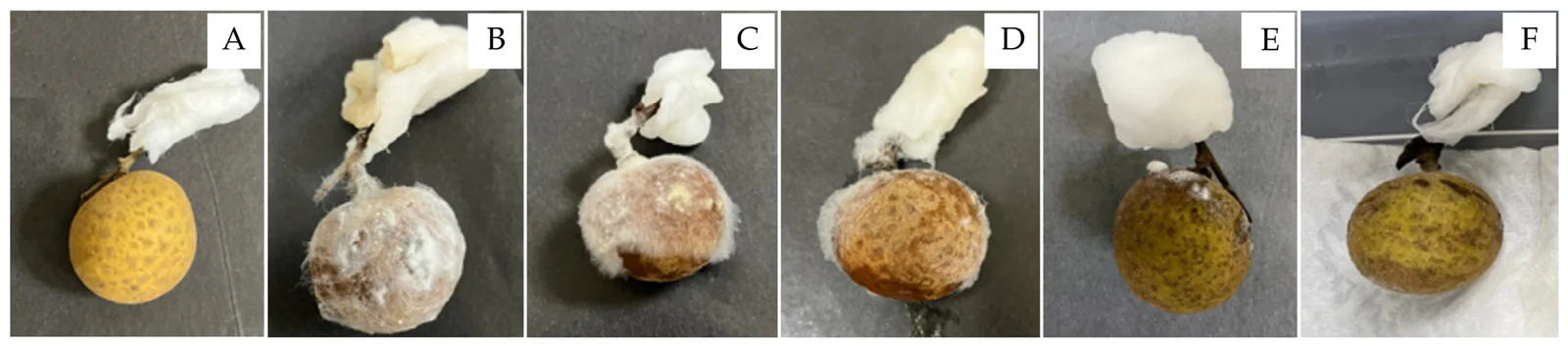
Incidence of longan fruit rot disease following combined inoculation with *B. amyloliquefaciens* (UPBA63) and *B. subtilis* (SBP04) at different concentrations. Treatments included (**A**) spraying with sterile distilled water (negative control); (**B**) inoculation with *L. pseudotheobromae* alone (positive control); and application of the bacterial consortium (*B. amyloliquefaciens* + *B. subtilis*) at concentrations of (**C**) 10^2^ CFU/mL; (**D**) 10^4^ CFU/mL; (**E**) 10^6^ CFU/mL; and (**F**) 10^8^ CFU/mL.

**Figure 8:**
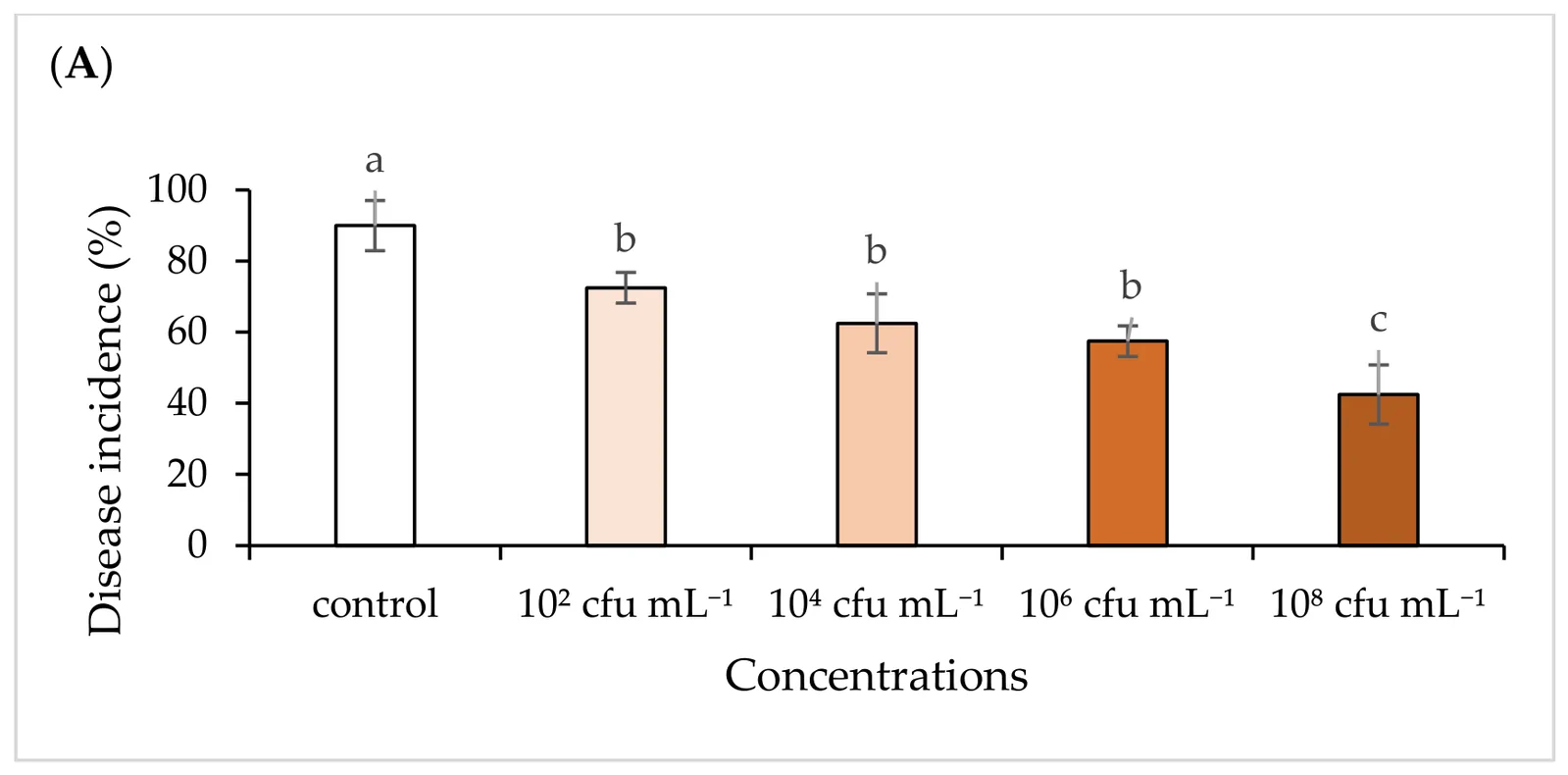

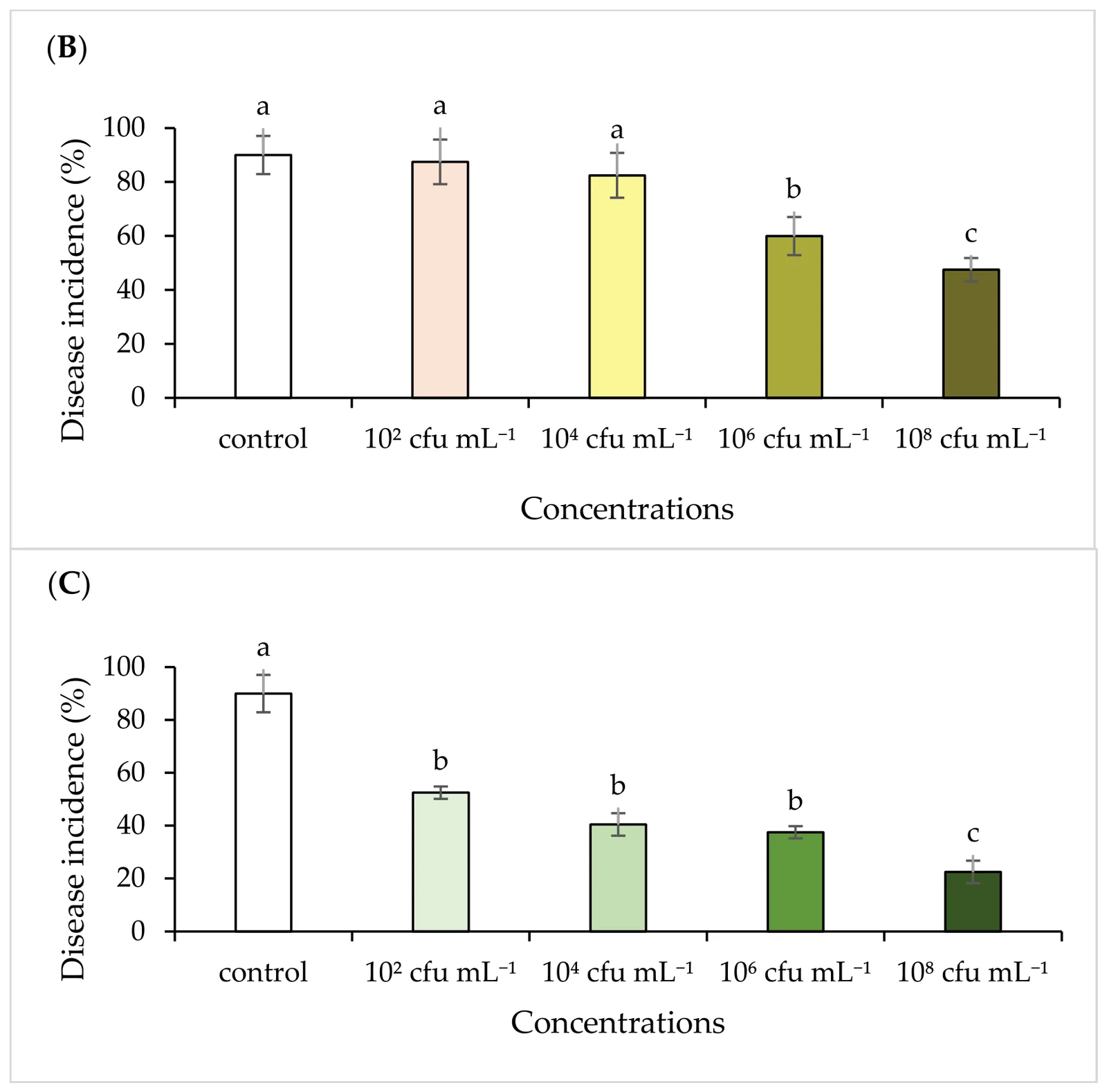
Incidence of longan fruit rot disease following treatment with different concentrations of (**A**) *B. amyloliquefaciens* (UPBA63); (**B**) *B. subtilis* (SBP04); and (**C**) a combined application of *B. amyloliquefaciens* + *B. subtilis* (UPBA63 + SBP04). Longan fruit inoculated with *L. pseudotheobromae* and sprayed with sterile water (without bacterial inoculation) served as the positive control. Data were analyzed using a one-way analysis of variance (ANOVA). Different lowercase letters indicate significant differences among isolates according to Tukey’s honestly significant difference (HSD) test at *p* < 0.05. Percentage data were arcsine square-root-transformed before analysis. Original (untransformed) means are presented.

The effect of the selected bacterial isolates on the incidence of disease caused by *L. pseudotheobromae* is presented in Figure 7. Plants inoculated with the pathogen alone, in the absence of bacterial inoculation, showed a high disease incidence of 90.00 ± 7.07%, whereas no disease symptoms were observed in the water-treated control. The application of bacterial isolates statistically significantly (*p* < 0.05) reduced disease incidence compared with that in the pathogen-only treatment. For all bacterial treatments, increasing bacterial concentrations resulted in a gradual reduction in disease incidence. At the lowest concentration (10^2^ cfu mL^−1^), disease incidence ranged from 52.50 ± 2.33% to 87.50 ± 8.29%, depending on the bacterial treatment. As the bacterial concentration increased to 10^8^ cfu mL^−1^, disease incidence decreased to 22.50 ± 4.29% in the combined inoculation treatment (UPBA63 + SBP04), which was the lowest among all treatments. Overall, the combined bacterial inoculation consistently exhibited strong disease suppression compared with single bacterial treatments across all concentration levels (Figure 8).

### 3.7. Evaluation of the Selected Phosphate-Solubilizing Bacterial Isolates Under Pot Conditions

Under *in planta* pot conditions, bacterial inoculation affected soil chemical properties, particularly available phosphorus. Prior to the experiment, soil samples were collected and analyzed. The soil had an average available phosphorus content of 109.13 mg kg^−1^, a pH of 4.35, and an organic matter (OM) content of 2.37%. After treatment, the control (distilled water) showed an available phosphate content of 107.92 ± 4.10 mg kg^−1^, which was similar to the initial value. Inoculation with the *B. amyloliquefaciens* isolate (UPBA63) slightly increased available phosphate to 114.50 ± 4.56 mg kg^−1^, whereas application of the *B. subtilis* isolate (SBP04) resulted in a greater increase to 118.67 ± 6.05 mg kg^−1^. The highest available phosphate, 146.92 ± 7.13 mg kg^−1^, was observed in the combined bacterial treatment (UPBA63 + SBP04 + organic matter), indicating a synergistic effect of the two isolates in enhancing phosphorus availability. In addition to increasing available phosphate, soil pH varied among treatments, with the combined treatment with organic matter showing the lowest, 5.20 ± 0.16, compared with the control, 5.42 ± 0.29 (Table 4).

### 3.8. Evaluation of a Multi-Strain Bacillus Formulation Under Field Conditions

Application of the multi-strain *Bacillus* formulation demonstrably influenced disease occurrence, soil characteristics, and longan tree productivity in a field setting, as detailed in Table 5 and Table 6. The control group exhibited a disease incidence of 47.38 ± 17.12%. Following bacterial inoculation, this incidence was substantially reduced, declining to 19.91 ± 7.22%. Furthermore, a further decrease to 15.40 ± 7.42% was observed when bacterial inoculation was coupled with a reduction in chemical inputs. Bacterial application also increased soil available phosphorus from 109.75 ± 5.52 mg kg^−1^ in the control to approximately 120.65 ± 7.42 to 149.25 ± 10.18 mg kg^−1^, while treatments receiving only chemical inputs showed lower values. In addition, bacterial treatments slightly increased soil pH and organic matter content. Longan yield was lowest in the control (15.92 ± 4.10 kg tree^−1^) and increased with bacterial inoculation (28.67 ± 5.99 kg tree^−1^), and the combination of bacterial inoculum with reduced chemical inputs produced yields (42.17 ± 6.29 kg tree^−1^) comparable to those obtained with the full chemical treatment (45.58 ± 8.95 kg tree^−1^).

## 4. Discussion

Longan production in northern Thailand faces serious threats from fruit rot and peel discoloration. Brown spot symptoms, with a disease incidence of up to 80%, have been reported in orchards in Phayao Province. *Lasiodiplodia pseudotheobromae* is considered a principal causal agent of fruit rot and continues to limit longan production in the region [15]. The high disease incidence implies persistent pathogen activity across the orchard, storage, and distribution chain, not merely opportunistic infection. Thus, effective control requires coordinated intervention during preharvest and postharvest phases. Current management practices, however, may fail to maintain stable protection during storage and distribution, particularly in production systems exposed to high humidity and prolonged transport. Although synthetic fungicide treatments can delay visible symptom development, stricter regulations on residues and growing concern over fungicide-resistant pathogen populations limit the long-term viability of chemical control.

Beneficial microorganisms have attracted considerable research attention for their capacity to support plant health, improve soil fertility, and promote environmentally responsible disease management [13,56,57,58]. Bacterial endophytes, particularly *Bacillus* species, are promising biocontrol candidates for combating destructive fungal pathogens [59,60]. Their antagonistic activity involves volatile organic compounds and antimicrobial metabolites, including mycosubtilins, iturins, and bacillomycins, produced by *B. subtilis* and related *Bacillus* species [61,62]. Owing to their antimicrobial properties, selected *Bacillus* strains can contribute to microbial biopesticide development for biological disease control [63,64]. Beyond pathogen suppression, numerous *Bacillus* species solubilize phosphate and convert poorly soluble soil phosphorus into plant-accessible forms, improving nutrient availability and promoting plant growth [65,66]. Phosphorus limitation is particularly severe in tropical agricultural soils and perennial orchards, where prolonged fertilizer use can lead to accumulation of poorly available phosphorus fractions [67]. Therefore, microbial inoculants able to both suppress pathogens and mobilize phosphorus may strengthen sustainable orchard production. Previous research has largely examined disease suppression or nutrient mobilization by *Bacillus* spp. separately [68,69], whereas limited work has evaluated both functions in perennial fruit crops, particularly in longan.

Moreover, the inhibition level recorded in the present study exceeded values reported for several *Bacillus* strains evaluated in comparable pot and field experiments, supporting a possible strain-specific advantage meriting mechanistic examination [70,71]. *Bacillus* spp. are widely recognized for their strong antifungal activity against diverse plant pathogens [72,73,74]. The selected *Bacillus* isolates exhibited hydrolytic enzyme activity during qualitative screening (Table 1 and Table 2), which may have contributed to their antagonistic effects. *Bacillus* spp. are also known to produce antimicrobial lipopeptides that suppress fungal pathogens; however, lipopeptide production was not assessed in this study. Volatile organic compounds have been reported to contribute to fungal inhibition in several *Bacillus* species [63,75]. Although VOC production was not evaluated in this study, it may represent one of the possible mechanisms underlying the observed antagonistic activity. Laboratory antagonism, however, cannot fully explain field efficacy, given reports of strong culture-based inhibition followed by limited disease suppression after application [76]. The results of the present study revealed variation in ACC deaminase production and in protease, amylase, and cellulase activities across the tested isolates, as presented in Table 1 and Table 2. Hydrolytic enzyme production by plant-growth-promoting rhizobacteria may promote recruitment of beneficial rhizosphere microorganisms associated with plant growth promotion and biological control [72,73,74,77]. Following bacterial establishment, colonization of potential infection sites may reduce pathogen entry. Competition for space and nutrients probably complements antimicrobial metabolite activity. The density-dependent suppression recorded in this study implies that sufficient accumulation of *Bacillus*-derived antimicrobial metabolites plays a critical role in effective inhibition of *L. pseudotheobromae*. Specific lipopeptides were not directly quantified, leaving the relative contribution of individual mechanisms unresolved.

Previous studies have reported that the combined application of *Bacillus subtilis* and *Bacillus amyloliquefaciens* can effectively suppress fungal diseases while promoting plant growth [78,79]. Consistent with these findings, the combined application of *B. amyloliquefaciens* (UPBA63) and *B. subtilis* (SBP04) in the present study reduced disease incidence and improved plant performance, supporting the potential of complementary *Bacillus* consortia for sustainable disease management. The pathogenicity assay revealed that plants inoculated with *L. pseudotheobromae* alone exhibited a high disease incidence (90%), indicating the strong virulence of the pathogen. Moreover, scanning electron microscopy (SEM) observations further revealed that the pathogen was able to penetrate the longan pericarp, in contrast to the uninoculated control, indicating its strong invasive capacity and its role in disease development [15,80,81]. This observation is consistent with the production of cell-wall-degrading enzymes, commonly associated with fungal pathogenicity [82,83].

The results showed that inoculation with the selected bacterial isolates significantly reduced disease development, particularly when the two *Bacillus* strains were applied together. The superior performance of the combined treatment across all bacterial concentrations suggests a synergistic interaction between the two *Bacillus* species. The antagonistic strains may inhibit pathogen invasion through multiple mechanisms, including the production of extracellular enzymes and antimicrobial compounds that inhibit fungal growth and infection processes [84,85]. Another possible explanation is the induction of plant defense responses, which has been reported for several *Bacillus* species. However, induced resistance was not investigated in the present study. In addition, synergistic interactions among plant-growth-promoting rhizobacteria have been widely reported, where multiple bacterial strains contribute complementary mechanisms that enhance biological control efficiency and ecological fitness in the rhizosphere [86,87,88]. In the in planta experiment, disease incidence declined from 90% in the pathogen control to 22.50 ± 4.29% at the highest bacterial concentration. Lower bacterial concentrations produced variable outcomes among sampling dates, while higher concentrations consistently reduced infection. Similar behavior has been reported for plant-associated bacteria in which effective exclusion of pathogens occurs only after microbial populations reach sufficient abundance to compete for infection sites [89,90]. In addition, available phosphorus increased from 109.13 mg kg^−1^ to 146.92 ± 7.13 mg kg^−1^ in the plots subjected to the combined bacterial treatment (UPBA63 + SBP04) (Table 4). This magnitude of change is within the range reported for phosphate-solubilizing bacteria in orchard soils [91,92,93,94].

The antagonistic activity of *B. amyloliquefaciens* UPBA63 against *L. pseudotheobromae* may derive from antifungal metabolites and hydrolytic enzymes. Earlier research documented production of iturins, fengycins, and surfactins—compounds capable of restricting fungal growth and disrupting pathogen cell membranes—by *Bacillus* spp. [84]. Metabolite production and enzymatic activity may account for the pathogen suppression recorded in the field trial. The *Bacillus*-based treatment achieved disease control and yield performance comparable to those obtained using full chemical treatment with a 50% reduction in agrochemical input. This comparable efficacy supports a possible synergistic interaction between the bacterial consortium and reduced fungicide application. A management program combining biological agents with lower fungicide doses may strengthen disease control efficiency and reduce chemical dependence in orchard production.

A microorganism selected for bioinoculant development should exhibit multifunctionality, employ multiple plant-growth-promoting mechanisms, and interact effectively with diverse crop species [95,96]. Beyond disease suppression, inoculation with UPBA63 and SBP04 improved several soil properties. Table 6 records a soil pH of 4.68 ± 0.26 and an organic matter content of 2.40 ± 0.25% following inoculation. Available phosphorus reached 120.65 ± 7.42 mg kg^−1^, representing the most pronounced response. Baseline available phosphorus was relatively high, though bacterial inoculation promoted greater phosphorus solubilization. It should also be noted that the initial soil contained a relatively high level of available phosphorus. Consequently, the beneficial effects of phosphate-solubilizing bacteria may have been less pronounced than would be expected under phosphorus-deficient soil conditions. Organic acids and phosphatases produced by phosphate-solubilizing bacteria may convert insoluble phosphorus compounds into plant-accessible forms, thereby improving nutrient availability.

In acidic soils, a considerable proportion of phosphorus may remain unavailable due to fixation by iron and aluminum compounds, despite relatively high soil phosphorus levels. The increase in available phosphorus following bacterial inoculation indicates that the isolate mobilized sparingly soluble phosphorus fractions, thereby increasing phosphorus availability beyond the initial soil level. Similar observations have been reported for phosphate-solubilizing *Bacillus* species, which release organic acids and phosphatases that facilitate phosphorus solubilization in soil [92,97]. Improved soil fertility may partly explain the enhanced yield observed in the bacterial treatment. Longan trees inoculated with the bacterial isolates combined with reduced chemical inputs produced yields of 42.17 ± 6.29 kg tree^−1^, which was more than double that of the untreated control (15.92 ± 4.10 kg tree^−1^). The increased fruit yield observed in the bacterial treatments may be attributed to improved nutrient availability and enhanced plant growth resulting from phosphate solubilization and phytohormone production. Previous studies have shown that plant-growth-promoting rhizobacteria can improve crop productivity by enhancing nutrient uptake, stimulating root development, and increasing plant vigor [87,98]. This result suggests that beneficial microorganisms not only contribute to disease suppression but also enhance plant growth through improved nutrient availability and soil properties.

The superior performance of the combined bacterial treatment may be attributed to the complementary functions of the two bacterial strains. *B. amyloliquefaciens* UPBA63 primarily contributed to pathogen suppression, whereas *Bacillus subtilis* SBP04 enhanced phosphorus availability and plant growth through phosphate solubilization and IAA production. In addition, organic matter may have provided a favorable environment for microbial establishment and activity in the rhizosphere, resulting in synergistic effects on plant growth and soil fertility [89,98]. In general, bioinoculants can be used in several ways. As seen throughout the text, the main method is to apply them directly to the soil, where they facilitate the mobilization of phosphorus that is otherwise unavailable to plants [94,99,100]. Moreover, the inoculants can be applied together with phosphate rocks to enhance phosphorus solubilization and improve phosphorus availability to plants [101].

The present findings indicate that selected *Bacillus* isolates possess biologically relevant activity against *L. pseudotheobromae*, together with phosphate-solubilizing capability. The study demonstrates that the beneficial effects observed under greenhouse and field conditions resulted from the complementary functions of the two *Bacillus* strains rather than equivalent dual functionality within each isolate. *B. amyloliquefaciens* UPBA63 primarily contributed to pathogen suppression, whereas *B. subtilis* SBP04 mainly enhanced phosphate solubilization and plant growth. Their combined application therefore provided an integrated strategy linking biological disease control with nutrient mobilization.

Although the integrated treatment containing the *Bacillus* consortium, organic matter, half-rate chemical fertilizer, and half-rate fungicide effectively maintained relatively low disease incidence under reduced chemical inputs (Table 5), the full chemical treatment remained the most effective in suppressing disease and achieving the highest fruit yield. Furthermore, the integrated treatment did not differ significantly from the corresponding treatment without the *Bacillus* consortium, indicating that the independent contribution of the *Bacillus* consortium could not be clearly distinguished from the effects of organic matter and reduced chemical inputs under the conditions of this field trial. Therefore, the present findings support the use of the *Bacillus* consortium as a complementary component of integrated disease management rather than as a complete replacement for conventional chemical control.

The present study demonstrated promising effects of bacterial inoculation on disease suppression, soil fertility improvement, and fruit yield, several limitations should be acknowledged. Only two isolates, *B. amyloliquefaciens* UPBA63 and *B. subtilis SBP04*, were evaluated under field conditions, while their long-term persistence, rhizosphere colonization, population dynamics, and interactions with native microbial communities remain unknown.

In addition, both isolates exhibited antagonistic and plant-growth-promoting traits; however, the specific antimicrobial metabolites and molecular mechanisms responsible for disease suppression were not investigated. Under the present experimental conditions, the selected isolates suppressed disease development when applied shortly after pathogen inoculation, suggesting their potential as an early post-infection biological treatment; however, their preventive efficacy before pathogen exposure, particularly under preharvest conditions, requires further evaluation. Future studies should therefore focus on quantitative enzyme assays, metabolite profiling, molecular characterization of biocontrol mechanisms, and broader screening of beneficial microorganisms. Strain-level biosafety should also be confirmed, including the absence of undesirable traits and assessment of potential risks associated with HCN production. In addition, long-term persistence, formulation stability, storage stability, environmental safety, regulatory compliance, and performance across multiple locations and growing seasons should be evaluated before commercial or large-scale field application.

## 5. Conclusions

The selected *Bacillus* strains exhibited antagonistic activity against *L. pseudotheobromae* and phosphate-solubilizing ability under the experimental conditions. In the field trial, the integrated treatment consisting of the isolates combined with organic matter, half-rate chemical fertilizer, and half-rate fungicide reduced disease incidence to 15.40 ± 7.42% and improved soil pH (4.56 ± 0.20), organic matter (2.92 ± 0.36%), and available phosphorus (149.25 ± 10.18 mg kg^−1^) compared with those of the untreated control. Disease incidence in this treatment was not significantly different from that of the corresponding integrated treatment without *Bacillus* (16.81± 6.91%), whereas the full chemical treatment produced the lowest disease incidence (2.26 ± 5.82%) and the highest yield. This result indicates that the selected *Bacillus* strains have potential for incorporation into integrated longan production systems to improve soil properties while supporting strategies for reducing chemical inputs.

## Figures and Tables

**Table 1:** Plant-growth-promoting and extracellular enzyme production of selected bacterial isolates.

Bacterial Isolate	Soluble Phosphorus (mg/L)	IAA Production (ug/mL)	Zinc Solubilization	Ammonia Production	ACCD Production	Siderophore Production
UPBA63	77.52	86	−	++	++	++
SBP04	90.12	69	−	+++	+++	+++

“−“: negative result; ++, moderate reaction; +++, strong reaction.

**Table 2:** Production of extracellular enzymes by bacterial isolates.

Bacterial Isolate	Production of Extracellular Enzymes		
	Protease	Amylase	Cellulase
UPBA63	+++	+++	+++
SBP04	++	+++	++

“++”, and “+++” indicate moderate, and strong enzyme production, respectively; ++, halo diameter 5–10 mm; +++, >10 mm.

**Table 3:** Biochemical profiles of the selected bacterial isolates.

Biochemical Test	Bacterial Isolate	
	UPBA63	SBP04
Shape	Rod	Rod
Gram reaction	Gram-positive	Gram-positive
Aerobe-anaerobe test	−	−
Endospore forming	+	+
Starch Hydrolysis	+	+
VP test	+	+
Citrate test	+	+
Indole test	−	−
Motility test	+	+
Catalase test	+	+

“+” indicates a positive reaction; “−” indicates a negative reaction.

**Table 4:** Phosphate solubilization by bacterial isolates under pot conditions.

Treatments	P (mgP/kg)	pH ^ns^	OM (%)
1. Control (distilled water)	107.92 ± 4.10 ^e^	5.42 ± 0.29	3.22 ± 0.20 ^e^
2. Organic matter alone	113.00 ± 4.13 ^de^	5.38 ± 0.21	3.55 ± 0.23 ^bcd^
3. Selected bacterial inoculum (UPBA63)	114.50 ± 4.56 ^de^	5.39 ± 0.19	3.35 ± 0.19 ^cde^
4. Selected bacterial inoculum (SBP04)	118.67 ± 6.05 ^cd^	5.30 ± 0.14	3.29 ± 0.26 ^de^
5. Selected bacterial inoculum (UPBA63+ SBP04)	122.75 ± 4.96 ^c^	5.26 ± 0.14	3.34 ± 0.24 ^cde^
6. Selected bacterial inoculum (UPBA63) + organic matter	123.08 ± 7.06 ^c^	5.28 ± 0.32	3.59 ± 0.23 ^bc^
7. Selected bacterial inoculum (SBP04) + organic matter	137.83 ± 5.94 ^b^	5.23 ± 0.26	3.78 ± 0.22 ^ab^
8. Selected bacterial inoculum (UPBA63+ SBP04) + organic matter	146.92 ± 7.13 ^a^	5.20 ± 0.16	3.92 ± 0.15 ^a^

Note: ^ns^: Not significant; Values are presented as mean ± standard deviation. Means followed by different letters within the same column are significantly different according to Tukey’s honestly significant difference (HSD) test at *p* < 0.05.

**Table 5:** Effects of *Bacillus* spp., organic matter amendment, and integrated management practices on disease incidence and yield of longan.

Treatments	Disease Incidence (%)	Yield (kg/Tree)
1. Untreated control	47.38 ± 17.12 ^a^	15.92 ± 4.10 ^e^
2. Organic matter alone	46.71 ± 12.67 ^a^	23.67 ± 6.19 ^d^
3. *Bacillus* spp. (UPBA63 + SBP04)	19.91 ± 7.22 ^b^	28.67 ± 5.99 ^cd^
4. *Bacillus* spp. (UPBA63 + SBP04) + organic matter	20.51 ± 3.59 ^b^	36.33 ± 5.82 ^bc^
5. *Bacillus* spp. (UPBA63 + SBP04) + organic matter + ½ chemical fertilizer + ½ fungicide	15.40 ± 7.42 ^b^	42.17 ± 6.29 ^ab^
6. Organic matter + ½ chemical fertilizer + ½ fungicide	16.81 ± 6.91 ^b^	40.25 ± 5.45 ^ab^
7. Organic matter + full chemical fertilizer + full fungicide	2.26 ± 5.82 ^c^	45.58 ± 8.95 ^a^

Note: Values are presented as mean ± standard deviation. Means followed by different letters within the same column are significantly different according to Tukey’s honestly significant difference (HSD) test at *p* < 0.05. Percentage data were arcsine square-root-transformed before analysis. Original (untransformed) means are presented.

**Table 6:** Effects of *Bacillus* spp., organic matter amendment, and integrated management practices on selected soil physicochemical properties in the upper soil layer beneath the longan canopy.

Treatments	P (mgP/kg)	pH	OM (%)
1. Untreated control	109.75 ± 5.52 ^c^	4.36 ± 0.20 ^c^	2.36 ± 0.18 ^b^
2. Organic matter alone	114.70 ± 5.34 ^bc^	4.71 ± 0.30 ^a^	2.88 ± 0.24 ^a^
3. *Bacillus* spp. (UPBA63 + SBP04)	120.65 ± 7.42 ^b^	4.68 ± 0.26 ^ab^	2.40 ± 0.25 ^b^
4. *Bacillus* spp. (UPBA63 + SBP04) + organic matter	146.25 ± 7.41 ^a^	4.64 ± 0.26 ^ab^	3.01 ± 0.42 ^a^
5. *Bacillus* spp. (UPBA63 + SBP04) + organic matter + ½ chemical fertilizer + ½ fungicide	149.25 ± 10.18 ^a^	4.56 ± 0.20 ^abc^	2.92 ± 0.36 ^a^
6. Organic matter + ½ chemical fertilizer + ½ fungicide	115.65 ± 7.04 ^bc^	4.60 ± 0.19 ^ab^	2.82 ± 0.35 ^a^
7. Organic matter + full chemical fertilizer + full fungicide	117.60 ± 7.10 ^b^	4.48 ± 0.16 ^bc^	2.81 ± 0.41 ^a^

Note: Values are presented as mean ± standard deviation. Means followed by different letters within the same column are significantly different according to Tukey’s honestly significant difference (HSD) test at *p* < 0.05. Soil samples were collected from the upper 0–15 cm soil layer beneath the canopy drip line after six months of treatment application.

## Data Availability

The data that support the findings of this study are available from the corresponding authors upon reasonable request.
